# Mechanistic aspects of the isomerization of *Z*-vinylic tellurides double bonds in the synthesis of potassium *Z*-vinyltrifluoroborate salts

**DOI:** 10.1186/1860-5397-4-9

**Published:** 2008-02-05

**Authors:** Hélio A Stefani, Rafael C Guadagnin, Artur F Keppler, Giancarlo V Botteselle, João V Comasseto, Carlos A Suganuma

**Affiliations:** 1 Faculdade de Ciências Farmacêuticas, Universidade de São Paulo, São Paulo, Brazil; 2 Departamento de Biofísica, Universidade Federal de São Paulo, São Paulo, Brazil; 3 Instituto de Química, Universidade de São Paulo, São Paulo, Brazil

## Abstract

Through direct transmetalation reaction of *Z*-vinylic tellurides with *n*BuLi was observed the unexpected isomerization of double bonds leading to potassium *E*-vinyltrifluoroborates salts in low to moderate yields. Using EPR *spin trapping* experiments the radical species that promoted the stereoinversion of *Z*-vinylic organometallic species during the preparation of potassium vinyltrifluoroborate salts was identified. The experiments support the proposed mechanism, which is based on the homolytic cleavage of the Te*n*Bu bond.

## Background

Boronic acids and boronate esters are the most commonly used derivatives in Suzuki-Miyaura cross-coupling reactions. Recently, Molander *et al.* [1] and our group [2] have explored the use of potassium organotrifluoroborate salts as an alternative to the usual organoboron reagents in alkenyl-alkenyl [3], aryl-aryl [4], alkenyl-alkynyl [5], and alkenyl-aryl [6] cross-coupling reactions.

Distinct from the most commonly explored hydrometallation reactions, the hydrotelluration of alkynes exclusively forms *Z*-vinylic tellurides [7]. Vinylic tellurides have the ability to undergo tellurium-metal exchange reactions with several different commonly used, commercially available, or easily prepared organometallic reagents, leading to *Z*-vinyllithiums and *Z*-vinylcyanocuprates. In reactions promoted by Pd or Ni, these compounds undergo stereospecific coupling with a wide range of organic species [8]. The vinylic organometallic species obtained in this way can also react with carbonyl compounds, α,β-unsaturated systems, or epoxides [9–11] with complete retention of the double-bond stereochemistry.

Taking advantage of the regio- and stereocontrol of the preparation of *Z*-vinylic tellurides [12], and of the unique features of the transmetallation with complete retention of the original double bond geometry, we report herein the synthesis of potassium vinyltrifluoroborate salts by means of the Te-Li exchange reaction. To the best of our knowledge, this is the first reported preparation of potassium *E*-vinyltrifluoroborate salts from *Z*-vinylic tellurides.

## Results and Discussion

**Scheme 1:**
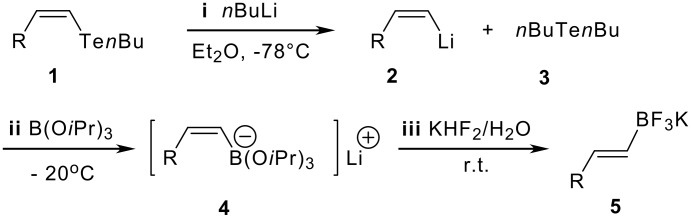
Synthetic route used to prepare vinyl BF_3_K salts.

Functionalized *Z*-vinylic tellurides **1** were prepared by hydrotelluration of alkynes [13]. Using phenyl vinyl telluride, we performed a series of test reactions to establish the best reaction conditions for the lithium-boron exchange step (Table 1; **ii**, Scheme 1). Optimum yield was obtained with B(O*i*Pr)_3_ as the electrophile and ether as the solvent (entry 6).

**Table 1:** Lithium-Boron Test Reaction Conditions.

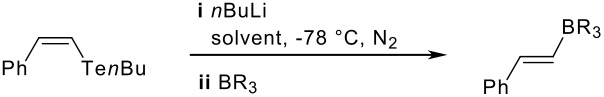			
**Entry**	**Electrophile (equiv)**	**Solvent**	**Yield (%)**

1	B(OMe)_3_ (1.5)	THF	18
2	B(O*i*Pr)_3_ (1.5)	THF	47
3	BF_3_.OEt_2_ (1.5)	THF	-
4	B(O*i*Pr)_3_ (1.5)	THF/HMPA	25
5	B(O*i*Pr)_3_ (1.5)	THF/TMEDA	-
6	B(O*i*Pr)_3_ (1.5)	Et_2_O	51
7	B(O*i*Pr)_3_ (1.5)	Et_2_O	15

**Figure 1:**
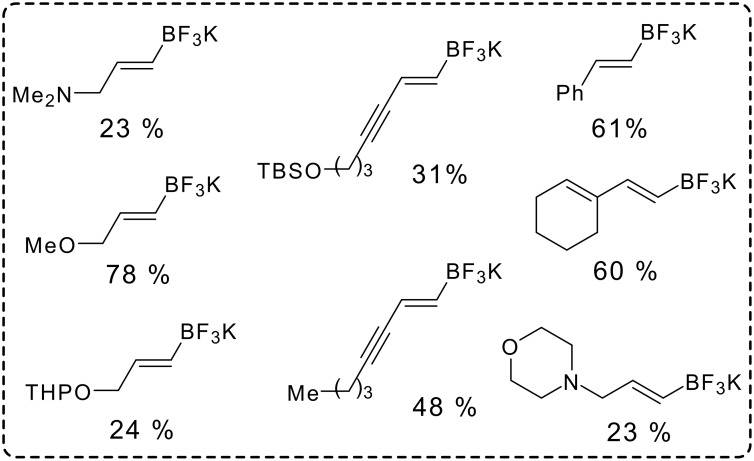
Isolated vinyl BF_3_K salts.

Using the optimized conditions (Table 1, entry 6), all the *Z*-vinylic tellurides were, to our surprise, transformed into potassium *E*-vinyltrifluoroborate salts exclusively (see Supporting Information File 1) (Figure 1).

The ^1^H NMR spectra of the products showed the presence of the salt *n*BuBF_3_K as a by-product (30–50% of the total yield). Use of 1.0 equiv of *n*BuLi instead of 1.5 equiv as in the optimized protocol gave the same proportion of *n*BuBF_3_K.

With ^1^H NMR, we tried to observe the coupling constants of the vinylic hydrogens for each intermediate of the reaction route. Using this approach, we could prove the *cis* geometry of the vinylic hydrogens of the intermediate **2** (Scheme 1), which presented a coupling constant of 18.09 Hz [14–15]. Unfortunately, the boronic “ate” complex **4** (Scheme 1) is an insoluble species and no ^1^H NMR spectra were obtained. However, these results indicated that the double bond geometry isomerization occurred only after the formation of the intermediate **4** (Scheme 1).

We suggest that homolytic cleavage of the Te-Bu bond, from **3** (**i**, Scheme 1), generates *n*Bu^•^, which is responsible for the *cis-trans* isomerization. The butyl radical attack occurs at the boronic “ate” complex **4** (Scheme 1) [16], yielding the *n*BuBF_3_K salt as a final product.

**Figure 2:**
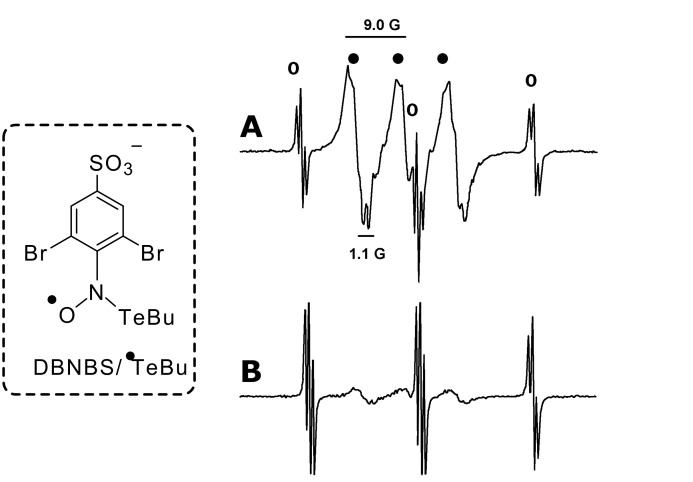
Representative EPR spectra of DBNBS radical adducts obtained during the Te-Li exchange reaction. (A) EPR spectrum obtained after 1 min incubation of the reaction mixture with the DBNBS aqueous solution, (B) EPR spectrum obtained after 15 min incubation of the reaction mixture with the DBNBS aqueous solution; (○) DBNBS/^•^TenBu radical adduct and (•) transient DBNBS radical adduct.

In order to verify the presence of radical species in the reaction mixture, we performed EPR spin trapping experiments using 3,5-dibromo-4-nitrosobenzenesulfonate (DBNBS), which is an appropriate spin trap for tellurium centered radicals [17]. Radical species were detected at the **i** and **ii** steps of the proposed route. In the first step (**i**, Scheme 1), the detected spectra contained a mixture of DBNBS radical adducts (Figure 2A). The triplet of triplets (*a*_N_ = 21.6 G, *a*_H_ = 0.7 G) is the DBNBS/^•^Te*n*Bu radical adduct [17] and the broadened triplet (*a*_N_ = 9.1 G, *a*_H_ = 1.0 G) can be attributed to another DBNBS radical adduct. The intensity of the broadened triplet started to decay after 5 min incubation, and was barely detected in the 15 min incubation spectrum (Figure 2B). The DBNBS/^•^Te*n*Bu signal maintained its intensity during the course of the EPR analysis.

**Figure 3:**
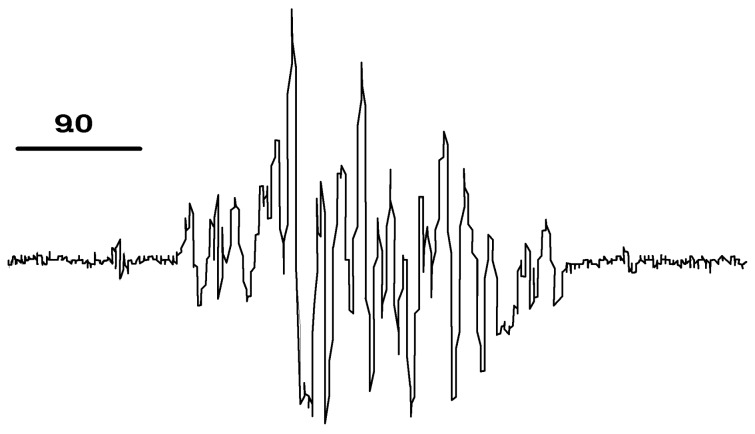
Representative EPR spectrum of DBNBS radical adducts obtained during the Li-Boron exchange reaction. EPR spectrum obtained after 15 min incubation of the reaction mixture with the DBNBS aqueous solution.

After the addition of the B(O*i*Pr)_3_ (**ii**, Scheme 1), the reaction mixture produced a complex EPR spectra that can be attributed to a mixture of radical species (Figure 3). The addition of the boron reagent generated different radical species from those observed in the previous reaction step (Figure 2).

We performed control experiments to exclude the possibility of radical generation by the combination of the boron reagent with O_2_ [18] or by the self-radical generation of the *n*BuTe*n*Bu reagent. Incubation of *n*BuTe*n*Bu, *n*BuLi and B(O*i*Pr)_3_ with DBNBS produced no EPR signals (Table 2, entries 3–5). Equimolar solutions of *n*BuTe*n*Bu, *n*BuLi and DBNBS (Table 2, entry 6) produced a radical signal with similar parameters as those detected during the Te-Li exchange (**i**, Scheme 1). In the absence of the reducing reagent (*n*BuLi), an equimolar solution of *n*BuTe*n*Bu, B(O*i*Pr)_3_ and DBNBS also did not produce EPR signals (Table 2, entry 8).

**Table 2:** Reactions and Control Experiments Performed.

**Entry**	**Reactions**	**DBNBS radical adducts EPR Hyperfines (G)**			
		a_N_	a_H_	a′_N_	a′_H_

1	BuTeCH=CHPh+*^n^*BuLi+DBNBS	21.6	0.7	9.1	1.0
2	LiCH=CHPh+B(O*^i^*Pr)_3_+DBNBS	complex signal			
3	BuTeBu+DBNBS	no signal			
4	*^n^*BuLi+DBNBS	no signal			
5	B(O*^i^*Pr)_3_+DBNBS	no signal			
6	BuTeBu+*^n^*BuLi+DBNBS	-	-	9.1	1.0
7	BuTeBu+*^n^*BuLi+B(O*^i^*Pr)_3_+DBNBS	complex signal			
8	BuTeBu+B(OiPr)_3_+DBNBS	no signal			

**Scheme 2:**
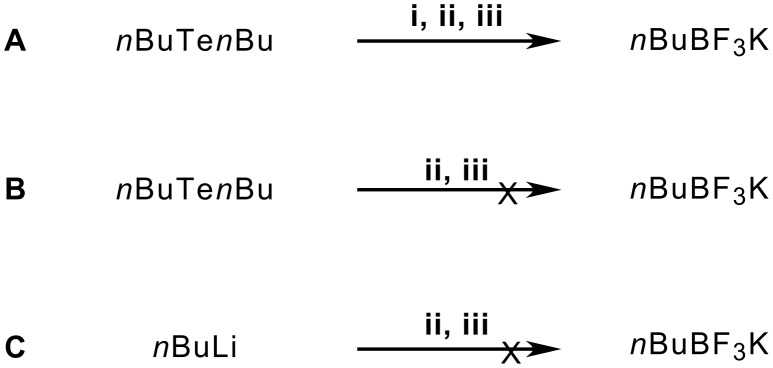
Experimental conditions. **i:** 1 equiv *n*BuLi, Et_2_O, −78 °C, 30 minutes. **ii:** 0.8 equiv B(O*i*Pr)_3_, −20 °C, 60 minutes. **iii.** 3 equiv KHF_2_ in aqueous solution, −20 °C to r.t., 30 minutes.

To test our proposed mechanism, we repeated the reaction using (Z)-β-bromostyrene, to achieve the desired *Z*-vinyllithium, the experiments were performed using *^t^*BuLi in a solution composed of THF/Et_2_O/petrol ether, at -120 °C, with and without *n*BuTe*n*Bu, instead of *Z*-vinylic tellurides to examine the effect of the *n*BuTe*n*Bu as the source of the butyl radical. From this reaction, the expected potassium vinyltrifluoroborate salt was not isolated, probably because it is necessary to use experimental conditions [19] that differ from those that were selected to perform the synthesis of the BF_3_K salts. To maintain the same reaction conditions, other control experiments were performed (Scheme 2).

Instead of having the double bond isomerization as a radical pathway model, evidence of *n*BuTe*n*Bu radical behavior came from the detection of *n*BuBF_3_K as a product only from experiment **A** (Scheme 2). With the control experiments (Scheme 2), it was proven that the generation of *n*BuBF_3_K salt is dependent on the presence of *n*BuTe*n*Bu, as well as that that occurs during the reaction to prepare the alkenyltrifluoroborate salts.

**Scheme 3:**
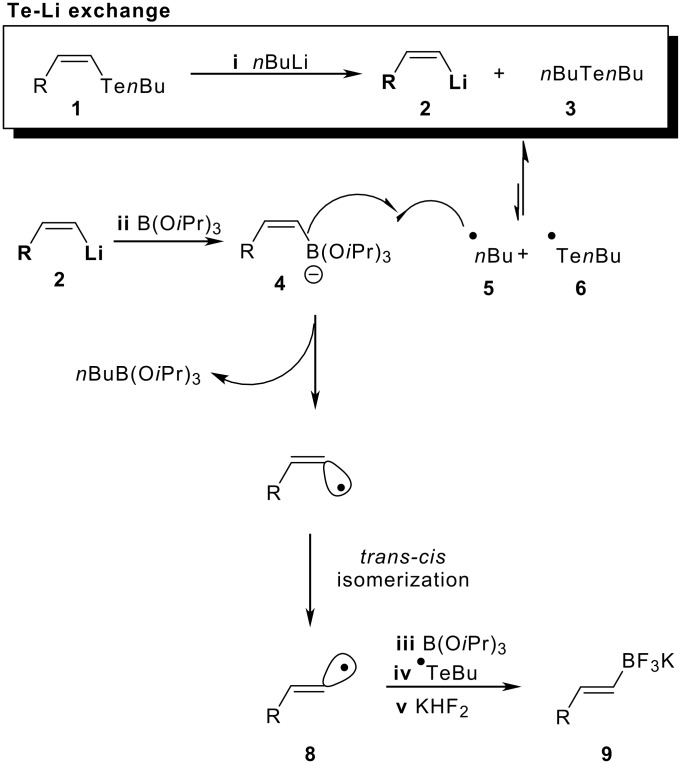
Proposed mechanism of the reaction.

The results presented above support a free radical pathway for the *trans-cis* double bond isomerization. Scheme 3 was proposed to account for the *E*-vinyl and *n*BuBF_3_K salts. In the first step, the butyl radical **5** is formed by homolytic cleavage of the *n*Bu-Te bond of the compound **3**, caused by the lithium species present in the reaction medium. The second step consists of an attack of **5** at the boronic “ate” complex **4**, leading to the vinylic radical, which undergoes self-isomerization to the most stable isomer **8**. In the third step, the vinylic radical **8** attacks a B(O*i*Pr)_3_ species, yielding an anionic vinyl boronic “ate” radical. The boron-centered radical is then reduced by a ^•^Te*n*Bu radical **6**, leading to the *E*-vinyltrifluoroborate salt **9** after the reaction work up with aqueous KHF_2_.

## Conclusion

In conclusion, we have identified the radical species that promoted the stereoinversion of vinylic compounds during the preparation of potassium vinyltrifluoroborate salts. The above experiments support the proposed mechanism, which is based on the homolytic cleavage of the Te*n*Bu bond.

## Supporting Information

File 1 Experimental section. The file describes the spectral data and the reaction procedure to prepare the potassium vinylorganotrifluoroborate salts
