# Supplementary material for: Mechanistic aspects of the isomerization of Z-vinylic tellurides double bonds in the synthesis of potassium Z-vinyltrifluoroborate salts
Source: Beilstein J Org Chem. 2008 Feb 5;4:9. doi: 10.1186/1860-5397-4-9 (PMC2244621; doi:10.1186/1860-5397-4-9)
Supplement: File 1 — Experimental section. The file describes the spectral data and the reaction procedure to prepare the potassium vinylorganotrifluoroborate salts [file Beilstein_J_Org_Chem-04-09-s001.doc]

**Experimental**

**General procedure to prepare potassium vinyltrifluoroborate salts synthesis of Z-vinylic tellurides:** *n*BuLi (12 mmol) was added dropwise at -70 ^o^C to a solution of the appropriated *Z*-vinylic telluride (10 mmol) in Et_2_O (30 mL). The bath temperature was raised to -20 ^o^C. After 20 minutes B(O*i*Pr)_3_ (15 mmol) was added at -40 ^o^C. After 1 hour, a aqueous solution of KHF_2_ (40 mmol in 15 mL of water) was added to the reaction mixture. Then, the solvent and water were eliminated under rotatory evaporator. To the obtained solid hot acetone was added and the bulk reactional was filtered

**(*E*)-Potassium styryltrifluoroborate salt**: Yield: 61%. ^1^H NMR (CDCl_3_, 300 MHz): δ 7.09-7.41 (m, 5H, Ph), 6.46 (d, JH-H = 18Hz, 1H), 6.18 (d, JH-H = 18Hz, 1H); ^13^C NMR (CDCl_3_, 75 MHz): δ 140.6, 133.54, 128.59, 126.19, 125.72.

**Potassium *n*-butyltrifluoroborate salt**: ^1^H NMR (CDCl_3_, 300 MHz): δ 1.18-1.03 (m, 5H, 2 CH_2_), 0.78 (t, 3H, CH_3_), 0.1-(-0,2) (m, 2H); ^13^C NMR (CDCl_3_, 75 MHz): δ 28.39, 26.31, 14.72.
